# Identifying Radical Pathways for Cu(I)/Cu(II) Relay Catalyzed Oxygenation via Online Coupled EPR/UV–Vis/Near‐IR Monitoring

**DOI:** 10.1002/advs.202402890

**Published:** 2024-05-29

**Authors:** Yongtao Wang, Yujia Zhou, Wenjing Sun, Xinyu Wang, Jia Yao, Haoran Li

**Affiliations:** ^1^ Department of Chemistry Zhejiang University 866 Yuhangtang Rd Hangzhou 310058 China; ^2^ Center of Chemistry for Frontier Technologies ZJU‐NHU United R&D Center Zhejiang University 866 Yuhangtang Rd Hangzhou 310058 China; ^3^ State Key Laboratory of Chemical Engineering and College of Chemical and Biological Engineering Zhejiang University 866 Yuhangtang Rd Hangzhou 310058 China

**Keywords:** alkyl hydroperoxide activation, alkylperoxyl radical, copper catalysis, Cu(II)‐alkoxyl radical complex, reaction mechanism

## Abstract

Copper‐catalyzed C─H oxygenation has drawn considerable attention in mechanistic studies. However, a comprehensive investigation combining radical pathways with a metal‐catalytic cycle is challenged by the intricate organic radicals and metallic intermediates. Herein, an online coupled EPR/UV–vis/near‐IR detecting method is developed to simultaneously monitor both reactive radical species and copper complex intermediates during the reaction. Focusing on copper‐catalyzed phenol oxygenation with cumene hydroperoxide, the short‐lived alkylperoxyl radical (EPR signal at *g* = 2.0143) as well as the unexpected square planar Cu(II)‐alkoxyl radical complex (near‐IR signal at 833 nm) are unveiled during the reaction, in addition to the observable phenoxyl radical in EPR, quinone product in UV–vis, and Cu(II) center in EPR. With a comprehensive picture of diverse intermediates evolving over the same timeline, a novel Cu(I)/Cu(II) proposed relay‐catalyzed sequential radical pathway. In this sequence, Cu(II) activates hydroperoxide through Cu(II)‐OOR into the alkylperoxide radical, while the reaction between Cu(I) and hydroperoxide leads to Cu(II)(•OR)OH with high H‐atom abstracting activity. These results provide a thorough understanding of the Cu(I)/Cu(II) relay catalysis for phenol oxygenation, setting the stage for mechanistic investigations into intricate radical reactions promoted by metallic complexes.

## Graphical abstract

To elucidate the mechanism of copper‐catalyzed C─H oxygenation, the online coupled EPR/UV–vis/near‐IR system is developed for simultaneous monitoring of both reactive radicals and metallic intermediates. The Cu(I)/Cu(II) relay catalysis is unveiled to enable the whole radical pathway: homolytic Cu─O cleavage of Cu(II)‐OOR releases ROO^•^ in Cu(II) catalysis, while the Cu(I) catalysis involves Cu(II)(^•^OR)OH with high H‐atom abstracting reactivity.

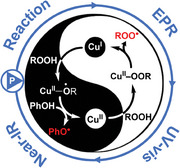

## 1 Introduction

**Scheme 1:**
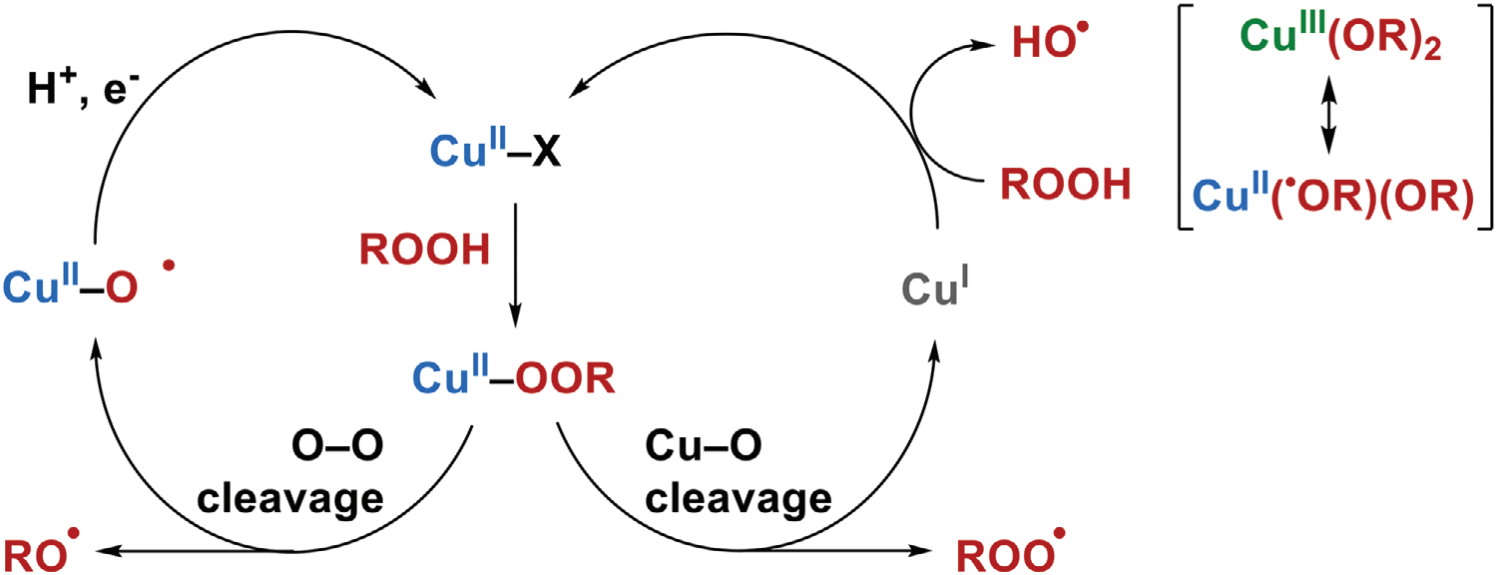
Mechanism for activation of hydroperoxides by mono‐copper centers.

Copper‐catalyzed C─H oxygenation widely exists in both living systems and industry processes, incorporating oxygen into C─H bonds.^[^ ^1^ ^]^ Hydroperoxides (H_2_O_2_ and ROOH) are commonly used in oxidative organic synthesis,^[^ ^2^ ^]^ and their activation by copper complexes involves diverse and reactive copper─oxygen complexes as well as O‐centered radicals.^[^ ^3^ ^]^ Despite various valence states of copper centers (Cu(I), Cu(II), or Cu(III)) being involved in one catalytic cycle, most proposed mechanisms incorporate only one type of oxygen activation for the entire reaction route. With limited examples, multi‐oxygen activations can be initiated by Cu(I), Cu(II), and even Cu(III) species within the same catalytic cycle, ultimately collaborating to achieve C─H oxygenation. Recently, a Cu(II)/Cu(III) relay catalysis has been proposed for alkane oxygenation with H_2_O_2_ as the oxidant,^[^ ^4^ ^]^ yet there is a lack of evidence for the involved O‐centered radicals and high‐valent Cu(III)‐OH intermediate. Utilizing cupric hydroperoxide (Cu(II)‐OOH) as the oxidant, Karlin, Solomon, et al. unveiled the Cu─O homolysis of Cu(II)‐OOH, followed by a Fenton‐type H_2_O_2_ activation with in situ formed Cu(I) toward Cu(II)‐OH and HO^•^ that actually results in oxidative N‐dealkylation reaction.^[^ ^5^ ^]^ The activation of hydroperoxides by Cu(I) or Cu(II) undergoes quite distinct mechanisms, thereby affording different reactive intermediates (**Scheme** **1**). It is challenging to elucidate relay catalysis involving multi‐valent copper centers in C─H oxygenation, due to the intricate radical and metallic intermediates.

The activation of hydroperoxides by Cu(II) normally generates cupric‐peroxo species, Cu(II)‐OOH or Cu(II)‐OOR,^[^ ^3^, ^6^ ^]^ where the alkalic ligands or additives are required for deprotonation of H_2_O_2_ or ROOH. Two homolytic cleavage mechanisms have been proposed for Cu(II)‐OOH(R) (Scheme 1): one is Cu−O cleavage that yields Cu(I) along with alkyl peroxyl radical (H(R)OO^•^) (Scheme 1, right cycle);^[^ ^5^ ^]^ the other is the O−O cleavage resulting in Cu(II)‐O^•^ with alkoxyl radical (R(H)O^•^) (Scheme 1, left cycle).^[^ ^6^, ^7^ ^]^ Via density functional theory (DFT) calculations, Karlin, Solomon et al. suggested Cu─O cleavage as the rate‐determining step during the reaction of Cu(II)‐OOH.^[^ ^5^ ^]^ Based on product analysis, Kitajima et al. also supported this cleavage during the decomposition of their Cu(II)‐OOCumyl complex.^[^ ^6a^ ^]^ Nonetheless, the O─O cleavage of Cu(II)‐OOR has been mostly proposed in C─H oxygenation,^[^ ^6^, ^7^, ^8^ ^]^ especially in the intramolecular copper‐directed hydroxylation.^[^ ^7b^ ^]^ It is quite challenging to distinguish between the two different pathways in catalytic reactions, because of the high reactivity and short lifetime of O‐centered radicals.^[^ ^9^ ^]^

On the other hand, the activation of hydroperoxides with Cu(I) species is generally suggested to undergo a Fenton‐type chemistry giving reactive HO^•^ and Cu(II)‐OH (or Cu(II)‐OR).^[^ ^10^ ^]^ Alternately, the oxidative addition of ROOR’ on Cu(I) has also been supported,^[^ ^11^ ^]^ affording Cu(III)‐centered intermediates. Extensive work about stabilized Cu(III) complexes has been reported,^[^ ^12^ ^]^ but it is still disputed whether the Cu(III) complex in solution persists in its Cu(III) core or turns to be Cu(II)‐radical species.^[^ ^11^, ^13^ ^]^ For instance, Storr et al. proposed the temperature relied on the equilibrium between Cu(III) and Cu(II)‐radical forms of the high‐valent copper complex.^[^ ^11a^ ^]^ Tolman et al. suggested a Cu(II)‐phenoxyl radical as the result of the reduction of their Cu(II)‐OOR complex;^[^ ^14^ ^]^ replacing phenoxyl ligand with alkoxyl, they also obtained Cu(III) complex.^[^ ^12g^ ^]^

In order to investigate the catalytic cycle involving multi‐valent copper centers, it is critical to track the time evolution of short‐lived radicals and metallic intermediates, then the combined analysis of the intermediates on the same timeline may give some clues. Recently, Rabeah et al. have introduced a combined EPR/UV–vis/ATR‐IR approach to investigate oxidation reactions,^[^ ^15^ ^]^ and showed its distinct advantages in a mechanistic study on copper/TEMPO catalyzed alcohol oxidation.^[^ ^15a^ ^]^ Inspired by their success, here we develop an online flowing method to simultaneously integrate EPR, UV–vis, and near‐IR detections at controlled temperatures. The temperature control allowed effective observation of reactive intermediates. Focusing on copper‐catalyzed phenol oxygenation using organic hydroperoxide as the oxidant, multiple signals were obtained with quantifiable intensity during the whole reaction, and control experiments, operando detections, spin capturing, as well as DFT calculations were conducted for their assignments. Via analysis of the simultaneous evolution of observed radicals and copper complex intermediates, a novel mechanism with Cu(I)/Cu(II) relay catalysis was unveiled.

## 2 Results and Discussion

### 2.1 Establish Online Coupled EPR/UV–Vis/Near‐IR System to Real‐Time Detect Copper Catalyzed Phenol Oxygenation

**Figure 1:**
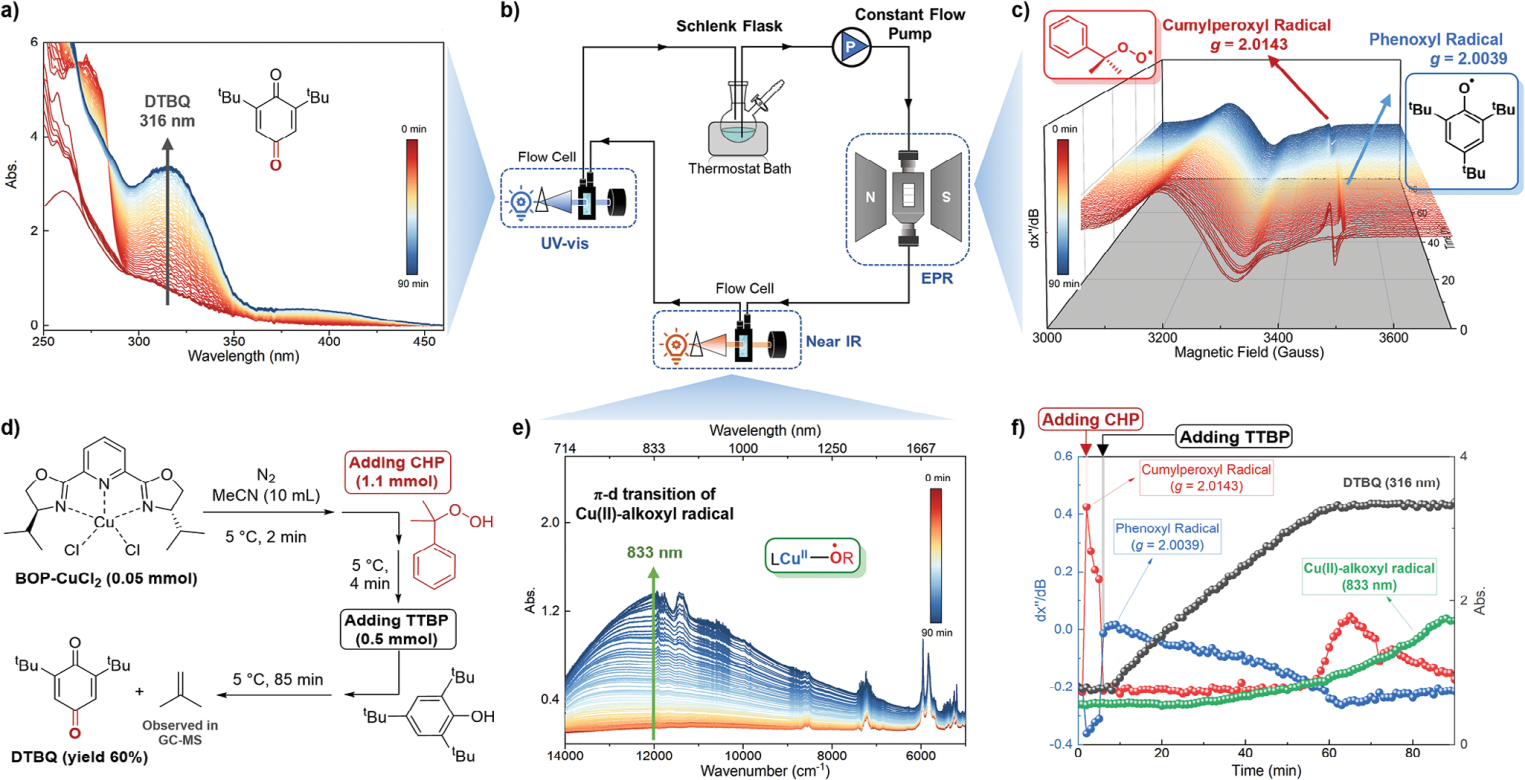
Time‐dependent spectra obtained with a) UV–vis, c) EPR (at frequency of 9.8480 GHz), ande) near‐IR spectroscopy. b) Schematic illustration of the online flowing system for coupled real‐time detection of EPR/UV–vis/near‐IR. d) Scheme of the whole reaction setup. f) Combined time course of different species observed via the coupled EPR/UV–vis/near‐IR on the same timeline.

An online flowing system connecting the reaction, EPR, UV–vis, and near‐IR spectrometers together is established to fulfill real‐time observing of copper‐catalyzed C─H oxygenation (see schematic diagram in **Figure** **1**b, and the physical picture is given in Supporting Information). The fast‐flowing solution through a thin tube enabled almost simultaneous and timely observation of the copper catalyst, radical intermediates, and the product, respectively, on the same reaction timeline. Notably, the flowing system makes it possible to easily control the reaction temperature, hence the short‐lived radical intermediates could be stabilized and observed by timely detections. Based on this system, the detailed mechanism through specific reactive oxygen species and complexes is anticipated for hydroperoxide activation by the copper complex.

**Scheme 2:**
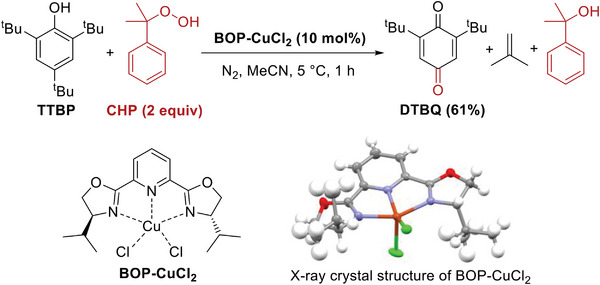
Mononuclear BOP‐CuCl_2_ catalyzed oxygenation of TTBP with CHP.

As a good H‐atom donor, the antioxidant 2,4,6‐tri‐*tert*‐butyl‐phenol (TTBP) is commonly used to verify the H‐atom abstracting ability of copper complexes,^[^ ^7^, ^12^ ^]^ giving observable phenoxyl radicals.^[^ ^16^ ^]^ Moreover, the oxygenation of TTBP is a typical reaction for mechanistic investigation of reactive oxygen species.^[^ ^17^ ^]^ Here we focus on TTBP oxygenation with cumene hydroperoxide (CHP) as the oxidant, employing a synthesized copper complex (BOP‐CuCl_2_) as the catalyst (**Scheme** **2**). The structure of the BOP‐CuCl_2_ complex was verified via ESI‐MS (Figure S1, Supporting Information) and X‐ray crystal characterization (Figure S3, Supporting Information). To conduct the reaction, a dosage of 2.2 equiv CHP was applied, and the reaction finished within 1 h at 5 °C, affording 2,6‐di‐*tert*‐butyl‐benzoquinone (DTBQ) in yield of 61%. In addition to 2‐phenyl‐2‐propanol derived from CHP, isobutylene was also confirmed to be one of the products (Figure S2, Supporting Information). Without a copper catalyst, no conversion was obtained under the same conditions.

To clarify how CHP is activated by the mononuclear copper catalyst and what is the catalytic cycle, the developed online monitoring system was applied for this reaction. As illustrated in Figure 1b, the MeCN solution of the BOP‐CuCl_2_ complex was stirred in a flask and maintained at 5 °C using a low‐temperature thermostat. The constant flow pump propelled the solution to flow rapidly through a 1 mm ID tube, connecting small‐volume optical flow cells for the determination of EPR, Near‐IR, and UV–vis in sequence. The flow time from each spectroscopy to another was less than 5 s, while that for a whole cycle was ≈10 s. For monitoring purposes, all three spectrometers collected spectra every minute. To initiate the reaction, the CHP and TTBP were separately added at 2 and 6 min, respectively (Figure 1d).

As expected, various species were quantitively observed by the three spectrometers in this monitoring system. In the UV–vis monitoring, though the absorption <260 nm was too strong to distinguish any species, an apparent peak emerged at 316 nm after the addition of TTBP (Figure 1a), which was attributed to the principal product, DTBQ, and was employed to quantify the overall reaction (Figure 1f, black points), indicating that the oxygenation finished within one hour. At the end period of DTBQ production, there emerged an intense absorption in near‐IR spectra, showing a broad peak ≈833 nm (Figure 1e). Considering the large wavelength and wide peak shape, it possibly belongs to Cu(II) or Cu(III) species, and our further studies suggested a Cu(II)‐alkoxyl radical complex for this signal (vide infra). Anyway, the absorbance at 833 nm was applied to quantify this species on the same timeline as that of DTBQ (Figure 1f, green points).

In EPR monitoring, several species were observed (Figure 1c). The Cu(II) complex in the solution exhibited a normal broad peak at a *g*‐value > 2.1, and the addition of CHP resulted in the shift of the Cu(II) peak to a smaller *g*‐value, indicating the change of the Cu(II) complex structure. Meanwhile, a signal at *g* = 2.0143 showed up as a new organic radical species. In response to the subsequent addition of TTBP, this signal disappeared instantly, while another signal at *g* = 2.0039 arose immediately. The EPR signal at *g* = 2.0039 gradually decreased accompanying the generation of DTBQ and faded away when the oxygenation finished. Therefore, it is reasonable to attribute this signal to the stable phenoxyl radical that derives from the H‐atom abstraction of TTBP. As a support, when TTBP was added into the BOP‐CuCl_2_ solution (Figure S4, Supporting Information), the signal at *g* = 2.0039 showed up, ruling out its possible assignment as CHP relative radicals. Moreover, DFT calculations confirmed the phenoxyl radical, giving the theoretical *g* value to be 2.0048.

The peak height values were used to quantify the two alternately appeared radical signals during the reaction and were shown in the same time course with DTBQ and Cu(II)‐alkoxyl radical complex (Figure 1f, red points for *g* = 2.0143 and blue points for *g* = 2.0039). The unknown signal at *g* = 2.0143 exhibited a highly reactive species toward the phenoxyl radical, disappearing at the beginning of the reaction and immediately appearing when the phenoxyl radical faded out. Therefore, the identification of this reactive radical intermediate is important to the whole mechanism. Following the above‐described online detections, additional control experiments, operando detections, spin capturing, and theoretical calculations were conducted to assign the unknown signals, then the mechanisms for CHP activation by Cu(II) and Cu(I) were discussed accordingly.

### 2.2 Evidences for Cu─O Cleavage in Cu(II)‐OOR Intermediate That Affords Reactive Alkylperoxyl Radical

**Figure 2:**
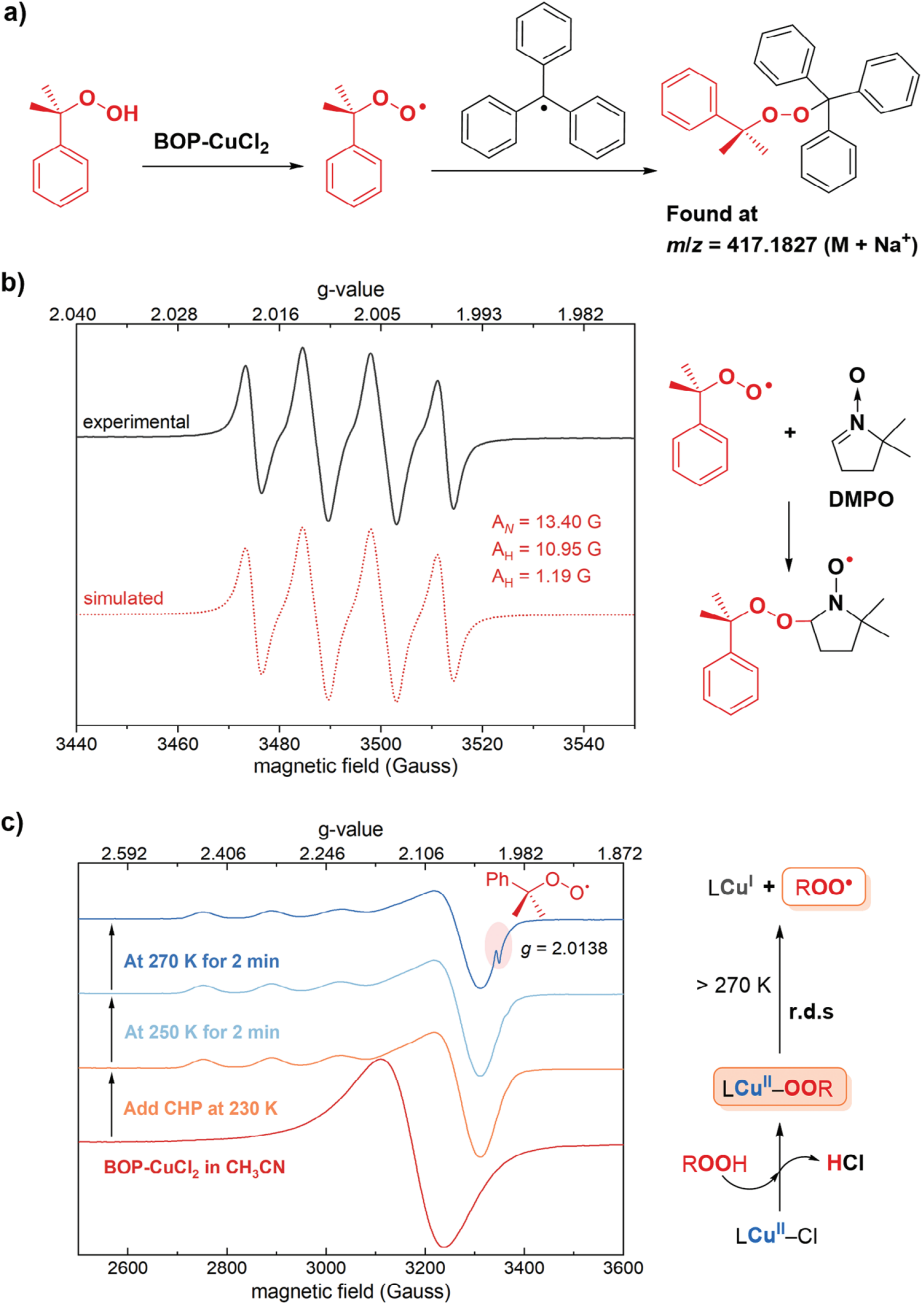
a) Capturing cumylperoxyl radical by trityl radical. b) Spin trapping of cumylperoxyl radical by DMPO at different reaction times. c) Sequent temperature control experiments detected by operando EPR, the spectra were collected at 200 K. Right: Activation of CHP by Cu(II) complex to generate cumylperoxyl radical.

The directly observed EPR signal located at *g* = 2.0143 can be tentatively assigned as cumylperoxyl radical because it immediately showed up after the addition of CHP into the BOP‐CuCl_2_ solution. This assignment is supported by DFT calculated *g* = 2.0130 for cumyl peroxyl radical. By the way, in the photolysis of di‐*tert*‐butyl peroxide in the presence of dioxygen, Fukuzumi et al. have reported the EPR signal of cumylperoxyl radical at *g* = 2.0156 in EtCN at −80 °C.^[^ ^9a^ ^]^ Two additional supports for this assignment came from the spin‐capturing experiments. One uses trityl radical and another uses 5,5‐dimethyl‐1‐pyrroline N‐oxide (DMPO). In trityl radical capturing, the triphenylmethyl bromide was introduced into the reaction solution to in situ generate trityl radicals and capture the possible cumylperoxide radical (**Figure** **2**a). To our delight, the coupled compound was observed in the capturing solution by high‐resolution mass spectrometry (HRMS) (Figure S5, Supporting Information), supporting the above assignment. DMPO could also capture the radical to form a more stable DMPO‐OOR radical that exhibits a characteristic splitting pattern in EPR spectra.^[^ ^18^ ^]^ It was therefore introduced as a spin trap into the reaction solution, and the following EPR detection afforded a well‐fitted signal with the coupling constants: *A*_N_ = 13.40 G, *A*_H_ = 10.95 G, and *A*_H_ = 1.19 G (Figure 2b). The obtained splitting pattern together with the fitted hyperfine coupling constants are in agreement with the typical DMPO‐OOR signal,^[^ ^19^ ^]^ enhancing the assignment of our directly observed signal at *g* = 2.0143 to cumylperoxide radical.

The mechanism for the generation of cumylperoxide radicals was then examined. Alkylperoxyl radical is mostly generated via the reaction between alkyl radical and dioxygen.^[^ ^9^, ^20^ ^]^ Under copper catalysis, limited work has suggested the homolytic Cu─O cleavage of Cu(II)‐OOH complex to release hydroperoxyl radical.^[^ ^5^ ^]^ Therefore, it is necessary to uncover how the cumylperoxyl radical is generated during the activation of CHP by BOP‐CuCl_2_. The operando EPR experiments were conducted under controlled temperatures to investigate the CHP activation mechanism in this work. To obtain the detailed signal with an anisotropic *g*‐tensor, the EPR spectra were collected using the frozen solution at 200 K. As shown in Figure 2c, the BOP‐CuCl_2_ catalyst in MeCN displayed a broad single peak at *g*_iso_ = 2.1235. Upon adding CHP into the solution and keeping it at 230 K for 10 min, the signal changed into a typical Cu(II) pattern with distinct g_//_ and g_⊥_, and no apparent radical signal was observed. The changed *g*‐tensor of the Cu(II) signal indicated that the structure of the Cu(II) complex is altered, leading to a different coordinating field. Increasing the temperature to 250 K for 2 min, there was no change in the signal. Nevertheless, when the temperature was increased to 270 K and maintained for 2 min, the Cu(II) signal turned weaker, accompanied by the emergence of a radical signal at *g* = 2.0138. This observed radical should be the cumylperoxide radical, consistent with that observed at *g* = 2.0143 during the reaction. Therefore, it can be proposed that the BOP‐CuCl_2_ complex easily reacts with CHP to afford the Cu(II)‐OOCumyl species, which turns reactive at higher temperatures than 270 K and generates both cumylperoxide radical and EPR‐silent LCu(I) complex via homolytic Cu─O cleavage (Figure 2c, right). Via electrospray ionization high‐resolution mass spectrometry (ESI‐HRMS) detection, the CuN_3_^+^ form of LCu(I) was suggested, with a couple of signals at *m/z* = 364.1076 and 366.1066 (Figure S6, Supporting Information). However, the CuN_3_Cl form of LCu(I) could not be totally excluded.

It has been well known that Cu(II) complex reacts with hydro‐ or alkylperoxide to form Cu(II)‐OOR(H), in the presence of HO^−^ or Et_3_N as proton acceptor.^[^ ^6^, ^14^ ^]^ Here the Cl^−^ in BOP‐CuCl_2_ may play the role to abstract the proton from CHP. Regarding the subsequent reaction of Cu(II)‐OOR(H), two radical pathways can be suggested: homolytic cleavage of Cu─O or O─O bond, respectively.^[^ ^6a^ ^]^ Although O─O cleavage has been predominantly suggested in previous reports,^[^ ^5^, ^7^, ^8^, ^10^ ^]^ here we demonstrated Cu─O cleavage in our experiments, aligning with that suggested by Karlin et al. via DFT calculations and carbon monoxide trapping experiments.^[^ ^5^ ^]^ In Karlin et al.’s work, the Cu−O cleavage was revealed to be the rate‐limiting step. Similarly, in this work, the generation of cumylperoxide radical from Cu(II)‐OOCumyl was also a slow step, occurring only when the temperature increased to 270 K. Overall, the activation of CHP by BOP‐CuCl_2_ was suggested to proceed through the homolysis Cu−O cleavage of the Cu(II)‐OOCumyl intermediate, based on the direct observation of cumylperoxide radical in both online monitoring and operando EPR detections.

### 2.3 Alkyl Hydroperoxide Activation by Cu(I)

As described above, the Cu(I) species was generated from the Cu─O homolysis of Cu(II)‐OOCumyl intermediate, thus the additional equivalent of CHP could be activated by Cu(I). Regarding previously reported reactions between Cu(I) and hydrogen peroxide (H_2_O_2_), the mostly suggested path is via a copper Fenton chemistry that affords Cu(II)‐OH and HO^•^ radical;^[^ ^5^, ^10^ ^]^ alternatively, the high valent Cu(III)(OH)_2_ is formed as the intermediate.^[^ ^11c^ ^]^ In our case, the signal of phenoxyl radical was observed throughout the entire oxygenation stage, only fading away when TTBP was consumed, after which the signal of cumylperoxide radical reemerged. Thus, the activation of CHP by Cu(I) may lead to the generation of the phenoxyl radical during the reaction, and the corresponding intermediate may accumulate and become evident when there is no TTBP in the solution.

**Figure 3:**
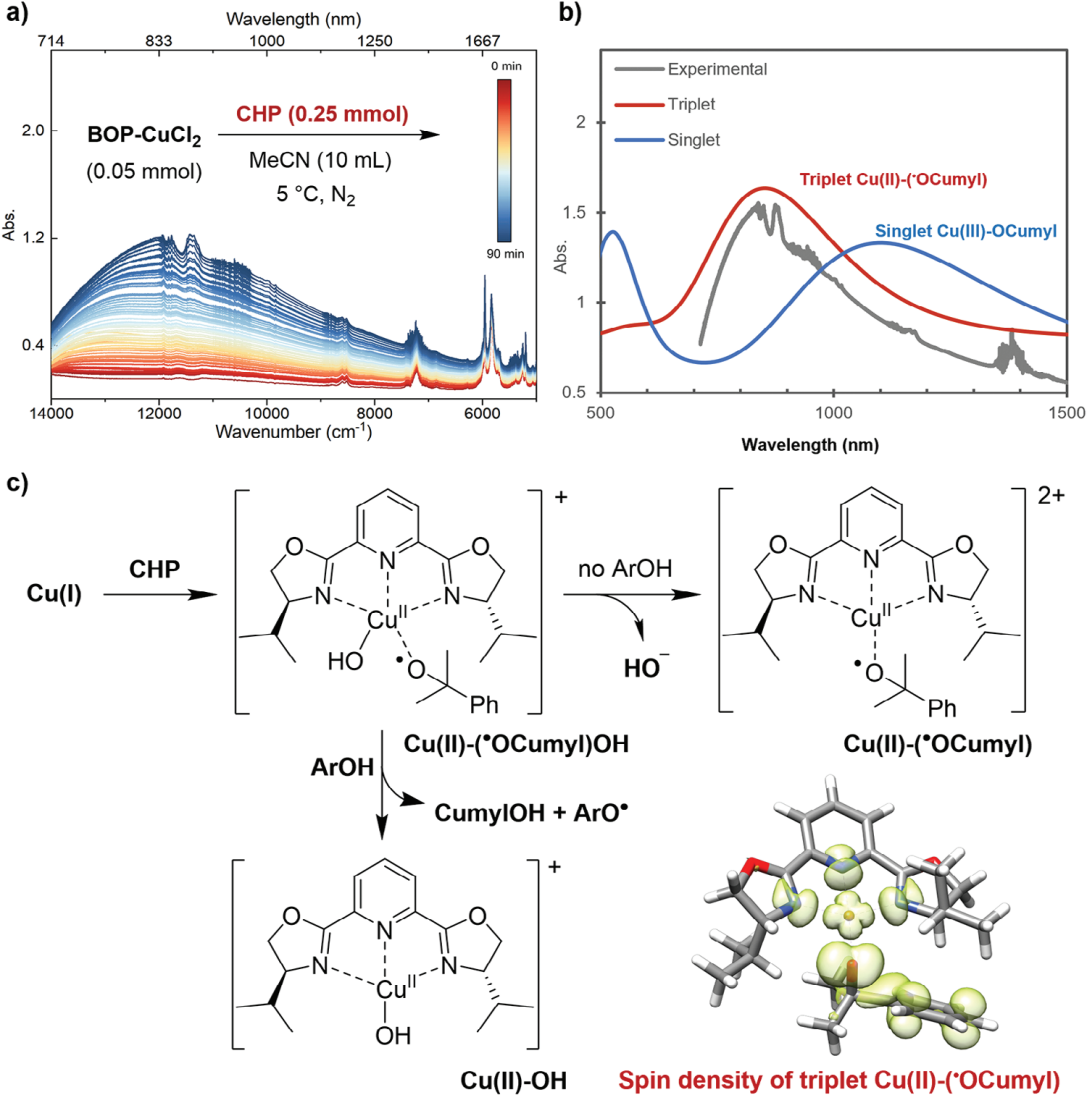
a) Time‐dependent spectra obtained with near‐IR spectroscopy. b) Experimental (grey) and TD‐DFT simulated near‐IR spectra (red for triplet and blue for singlet state). c) Formation and derivation of triplet Cu(II)(^•^OR)OH intermediate. Insert: Spin distribution plot of triplet Cu(II)‐(^•^OR) complex.

In the later stages of TTBQ generation, the near‐IR signal at ≈833 nm emerged and became stronger when TTBP was depleted (Figure 1e,f), providing a potential trace of the reactive intermediate derived from CHP activation by Cu(I). The broad‐peak shape signal should belong to high valent copper species. In previous work on high valent copper complexes, a similar broad signal has been attributed to the *π*→*d*(x^2^‐y^2^) transition of square planar Cu(III)‐OH,^[^ ^12^, ^21^ ^]^ Cu(III)‐OR,^[^ ^12g^ ^]^ Cu(III)‐X(F/Cl/Br),^[^ ^12e^ ^]^ or the Cu(II)‐phenoxyl radical complexes.^[^ ^14^ ^]^ In our case, the observed near‐IR peak could be attributed to either Cu(II)‐radical or Cu(III) formed from the reaction of Cu(I) with CHP. As a supporting observation, when CHP was added into the BOP‐CuCl_2_ solution in the absence of TTBP, a gradual increase in the same near‐IR signal was noted after the generation of cumylperoxide radical (**Figure** **3**a). Therefore, it is reasonable to assume the involvement of high‐valent copper intermediate rather than the Fenton‐type chemistry for the reaction of Cu(I) with CHP in our system.

This assumption is further supported by TDDFT calculations on the intense transition at 833 nm. Without a vacant *d* orbital, no signal at the near‐IR region will be found for Cu(I) complexes. Considering high‐valent copper species, the theoretical transitions of the triplet Cu(II)‐(^•^OCumyl) complex accurately replicated the experimental absorptions in the near‐IR region (Figure 3b, red). Conversely, the singlet Cu(III)‐OCumyl complex failed to match the experimental data (Figure 3b, blue), suggesting that the observed near‐IR signal originates from Cu(II)‐(^•^OCumyl) rather than Cu(III)‐OCumyl. Moreover, the spin distribution in triplet Cu(II)‐(^•^OCumyl) confirmed its cupric‐radical electronic structure (Figure 3c). Nevertheless, the Gibbs free energy difference between triplet Cu(II)‐(^•^OCumyl) and singlet Cu(III)‐OCumyl is as small as 0.2 kcal/mol, so it is hard to completely exclude the existence of Cu(III)‐OCumyl in our system.

Subsequently, the reactivity of the proposed Cu(II)‐(^•^OCumyl) complex was examined. When the near‐IR peak at 833 nm increased, TTBP was added to the solution. Nonetheless, there was no apparent decrease of the Cu(II)‐(^•^OCumyl) signal, indicating that it is not the intermediate responsible for oxidizing TTBP to phenoxyl radical during the reaction. Thus, the observed Cu(II)‐(^•^OCumyl) complex might be the side‐product of reactive Cu(II)(^•^OCumyl)OH or Cu(III)(OCumyl)OH complex formed by Cu(I) and CHP. Notably, the triplet Cu(II)(^•^OCumyl)OH was preferred by our DFT optimizations rather than the singlet Cu(III)(OCumyl)OH. We assume that during the reaction, the Cu(II)(^•^OCumyl)OH intermediate performed H‐atom abstraction on TTBP; in the absence of TTBP, it released hydroxyl anion to give rise to a more stable square planar Cu(II)‐(^•^OCumyl) (Figure 3c).

### 2.4 Cu(I)/Cu(II) Relay Catalyzed Sequential Radical Pathway

**Figure 4:**
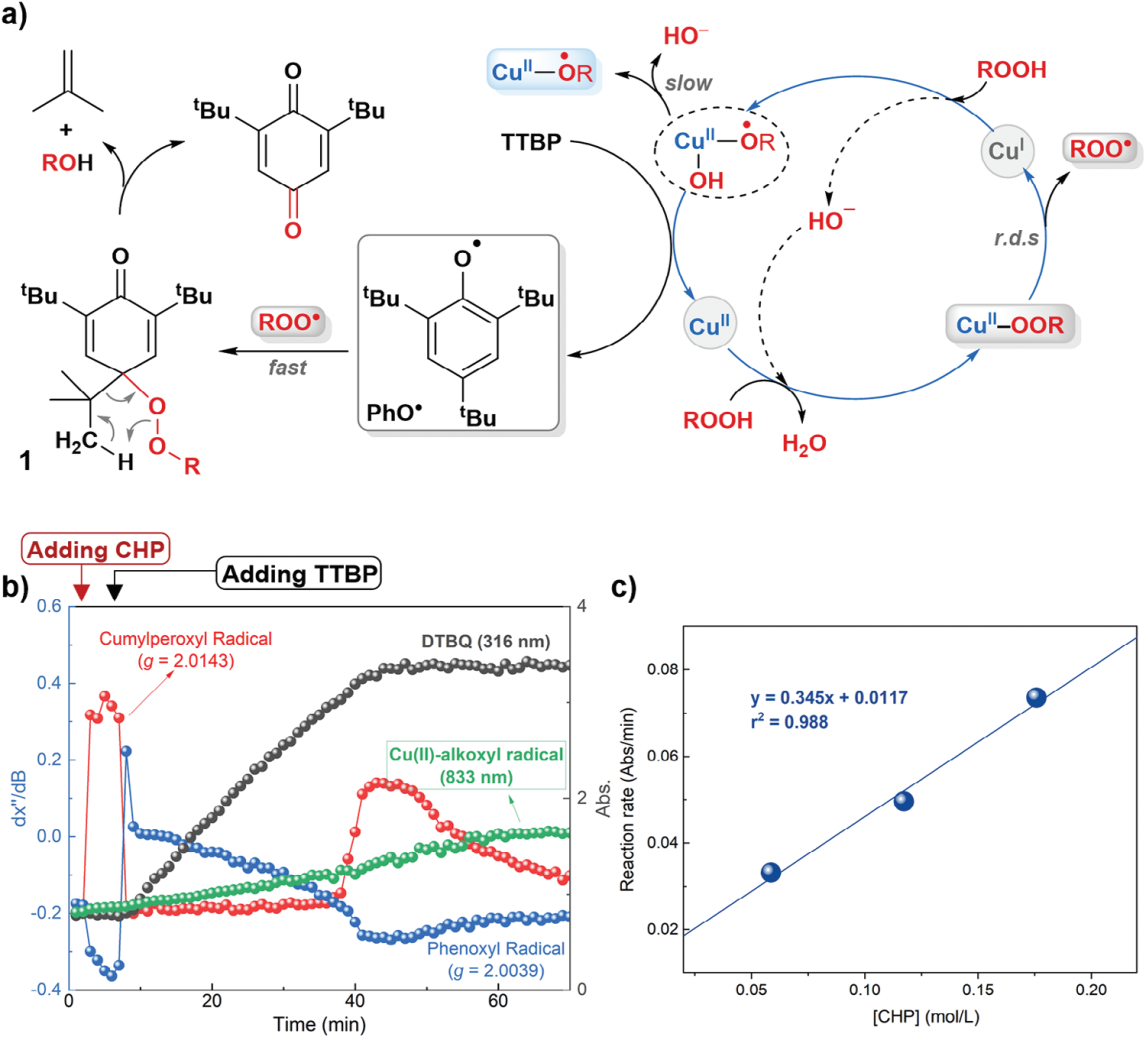
a) Proposed overall reaction mechanism. b) Combined time course of different species with 3.3 equivalent CHP added. c) Linear correlating of initial reaction rate with CHP concentration.

The almost simultaneous detections with UV–vis, near‐IR, and EPR in this online system allowed the time evolution of these active intermediates during the reaction to be quantitatively tracked (Figure 1f). With the combined time course for various species, we proposed the whole reaction mechanism as depicted in **Figure** **4**a. When adding CHP into the solution of BOP‐CuCl_2_ at 2 min, the immediately formed LCu(II)‐OOCumyl was active enough at 5 °C to generate the cumylperoxide radical together with EPR‐silent LCu(I) species, which could activate another equivalent CHP to form the Cu(II)(^•^OCumyl)OH intermediate. At 6 min, TTBP was introduced into the solution and easily reacted with Cu(II)(^•^OCumyl)OH to afford phenoxyl radical and Cu(II) species. The formed Cu(II) species reacted with CHP to yield Cu(II)‐OOCumyl for another catalysis cycle. The sharply decreased signal at *g* = 2.0143 indicated fast coupling between cumylperoxide radical and phenoxyl radical, giving rise to the singlet adduct **1**. As a support, the coupling between Cu(II)‐OO^•^ or Co(III)‐OO^•^ and TTBP phenoxyl radical have also been reported to generate MOO‐ArO complexes,^[^ ^17^, ^22^ ^]^ which then convert to DTBQ and isobutylene. Similarly, adduct **1** preferred to undergo the same reaction and eventually yield DTBQ together with isobutylene and cumyl alcohol.

To explore the reaction initialized by the Cu(I) complex, the Cu(I) species was prepared in situ by mixing CuCl with the BOP ligand, and then was applied as a catalyst in the reaction (Figure S8, Supporting Information). When adding CHP into the Cu(I) solution, the Cu(II) signal immediately arose in EPR spectra together with the cumylperoxyl radical. Therefore, unlike the gradually released Cu(I) species during the reaction, the formed Cu(II)‐(•OCumyl)OH species in Cu(I) solution may turn to react with another Cu(I) and generate Cu(II). Moreover, the Cu(I) initialized reaction resulted in a faster reaction at the beginning period, then extensive phenoxyl radical appeared at the middle period. Finally, though the reaction route was similar to that initialized by Cu(II), a decreased yield of quinone was obtained to be 53%, where the high concentration phenoxyl radical may lead to more side reactions.

Afterwards, the rate‐limiting step was considered. Employing the 3.3 equivalent of CHP instead of the 2.2 equivalent (Figure 4b; Figure S10, Supporting Information), a faster generation of DTBQ was observed, and both the cumylperoxide radical and Cu(II)(^•^OCumyl)OH appeared at an earlier time, matching the above‐proposed mechanism. The temperature‐control EPR detections suggested a slower Cu−O breaking than Cu(II)‐OOCumyl formation (Figure 2c). Therefore, attributing CumylOO^•^ generation from Cu─O cleavage to the rate‐determining step makes sense here. The real‐time detection enabled kinetic analysis, and the DTBQ generation rate was fitted according to the time course. A linear correlation between the obtained reaction rate and the initial CHP concentration is observed (Figure 4c), suggesting that the rate‐limiting step is the Cu─O cleavage of LCu(II)‐OOCumyl with one CHP molecule involved. Moreover, the reaction at room temperature (23 °C) was investigated with the same method, and the results are given in Figure S11 (Supporting Information). The intensity maximum of observed cumylperoxyl radical was quite lower than that at 5 °C, and the reason may be its worse stability at higher temperature. As a result, the reaction turned significantly slower at 23 °C, and the final yield of quinone decreased from 61% at 5 °C to 49% at 23 °C. Moreover, as the reaction proceeded, the intensity of phenoxyl radical increased significantly, which may be caused by the side reaction at higher temperature.

## 3 Conclusion

In summary, the online coupled EPR/UV–vis/near‐IR detecting system provided direct spectroscopic observation of diverse short‐lived radicals and metallic intermediates on the same timeline. Based on this online system, the whole picture of the mechanism for copper‐catalyzed oxygenation, including a Cu(I)/Cu(II) relay catalysis, can be obtained. When Cu(II) reacts with CHP, the alkalic ligand benefits to the heterolysis of O─H bond to form Cu(II)‐OOCumyl complex, whose homolytic Cu─O cleavage was revealed to release active CumylOO^•^ radical together with Cu(I) species. As to the reactivity of Cu(I), the homolysis of O─O bond was suggested in CHP to form Cu(II)(^•^OCumyl)OH intermediate that is able to oxidize phenol to phenoxyl radical and generate Cu(II) species. The overall Cu(I)/Cu(II) relay catalyzed radical pathway resulted in the selective oxygenation to afford quinone product. Without phenol, the Cu(II)(^•^OCumyl)OH may release hydroxyl anion to give the square planar Cu(II)‐(^•^OCumyl) as a stable species in the solution, showing a near‐IR signal at ≈833 nm. We believe the findings described here significantly advance the understanding of copper‐catalyzed oxygenation reactions, and the developed online coupled multi‐spectroscopy method can be widely applied to elucidate other complicated radical‐involved pathways.

## Conflict of Interest

The authors declare no conflict of interest.

## Supporting information



Supporting Information

## Data Availability

The data that support the findings of this study are available in the supplementary material of this article.
