# Supplementary material for: Identifying Radical Pathways for Cu(I)/Cu(II) Relay Catalyzed Oxygenation via Online Coupled EPR/UV–Vis/Near‐IR Monitoring
Source: Adv Sci (Weinh). 2024 May 29;11(29):2402890. doi: 10.1002/advs.202402890 (PMC11304242; doi:10.1002/advs.202402890)
Supplement: Supplementary file 1 — Supporting Information [file ADVS-11-2402890-s001.docx]

Supporting Information

Identifying Radical Pathways for Cu(I)/Cu(II) Relay Catalyzed Oxygenation via Online Coupled EPR/UV-vis/Near-IR Monitoring

Yongtao Wang,^†^ Yujia Zhou,^†^ Wenjing Sun, Xinyu Wang, Jia Yao, and Haoran Li*

1. Experimental Section

*General Considerations*:

All reagents were purchased from commercial sources and used without further purification unless otherwise noted. Gas chromatography (GC) was performed on GC-2014 (Shimadzu, Japan) with flame ionization detector (FID) and HP-1 MS column. Gas chromatography-mass spectrometry (GC-MS) spectra were obtained on a Shimadzu GC/MS-QP2010 system (Shimadzu, Germany). The high-resolution mass spectrometry (HRMS) spectra were obtained on Agilent 6545 Q-TOF-MS. X-ray single crystal diffraction were obtained on Bruker D8 Venture Ims3.0 (Bruker, Germany). Electron paramagnetic resonance spectrum (EPR) were obtained on Bruker EMXPlus-9.5/12 (Bruker, Germany). UV-vis spectroscopy was obtained on Shimadzu UV-2700i with a 0.5 mm Starna flowing cell. Near-IR spectroscopy was obtained on Thermo Scientific™ Antaris II, and the Starna flowing cell (1 mm) was used.

*Synthesis of BOP-CuCl_2_ complex*:

In a 50 mL round bottom flask, equivalent amounts of CuCl_2_ (2 mmol) and BOP ligand ((S,S)-2,6-bis(4-isopropyl-2-oxazolin-2-yl)pyridine, 2 mmol) were dissolved in acetonitrile (10 mL). After the mixture was stirred for 2 hours at room temperature, add diethyl ether dropwise until white precipitates appeared and slowly dissolved. Then put it in the freezer layer to crystallize. After precipitation of crystals, pour out the upper solution, wash with diethyl ether, and dry under vacuum at 30 °C, then 0.74 g product was obtained with a yield of 85%. HRMS (ESI) *m*/*z*: [M - H]^+^ calcd for C_17_H_23_Cl_2_CuN_3_O_2_, 399.0769; found, 399.0771.

CCDC 2310077 contains the supplementary crystallographic data for this paper. These data can be obtained free of charge from The Cambridge Crystallographic Data Centre via www.ccdc.cam.ac.uk/data_request/cif.


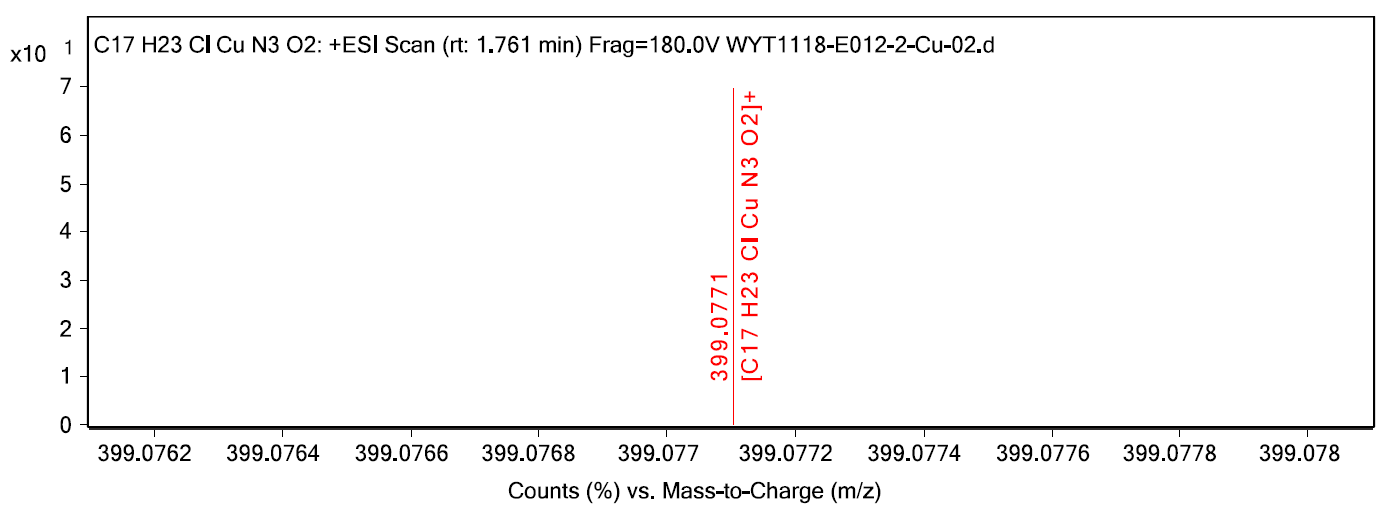


**Figure S1.** High resolution mass spectrum of [M-Cl]^+^ for BOP-CuCl_2_ in ESI-MS measurement.

*General procedure for oxidation*:

In a typical procedure for 2,4,6-tri-tert-butyl-phenol (TTBP) oxidation, a dried pressure resistant glass sample bottle (40 mL) was charged with 0.5 mmol TTBP and 0.05 mmol BOP-CuCl_2_ dissolved in 10 mL acetonitrile, and then was evacuated and filled with nitrogen. The tube was stirred in 5 °C, and the reaction was started by injecting about 0.2 mL 80% cumene hydroperoxide (CHP, 1 mmol) into the solution, then react for 1 hour at 5 °C. The conversion and yield were determined using gas chromatography with methyl benzoate as the internal standard, where the standard quinones were used to provide the standard curves. Isobutene was determined by GC-MS.


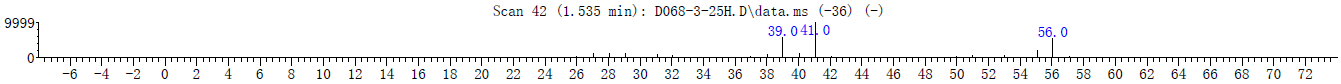


**Figure S2.** (a) Gas chromatography of general procedure for oxidation. (b) Mass spectrum of isobutylene obtained in GC-MS measurement.

*Online Coupled EPR/UV-vis/Near-IR Detections*:


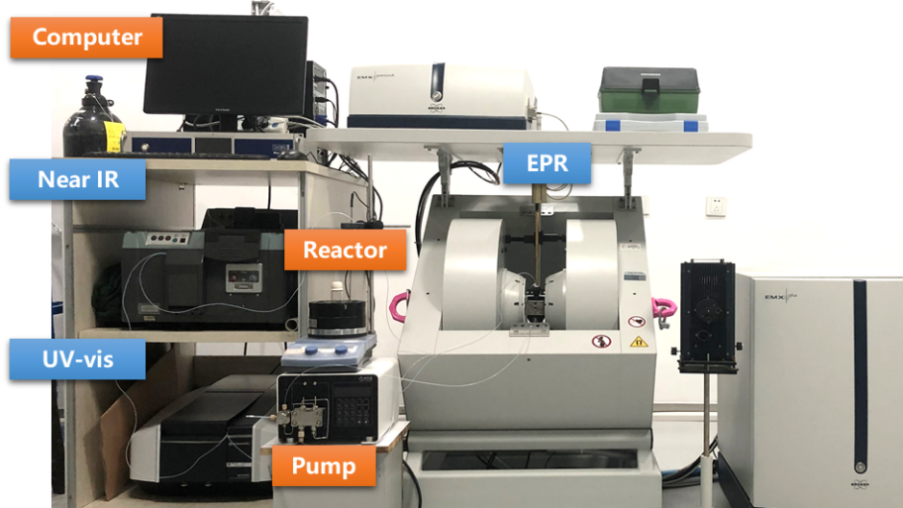


The online coupled EPR/UV-vis/Near-IR Detection was performed with the developed system above, built on basis of the designing depicted in Figure 1b. As a typical detection, add 0.05 mmol of BOP-CuCl_2_ to a 40 mL pressure resistant glass sample bottle, dissolve it in 9 mL of acetonitrile, and 0.5 mmol of TTBP dissolved in 1 mL of acetonitrile to another 20 mL bottle. Cover them with stoppers, and then vacuum and fill them with nitrogen gas. Open the spectral instrument and corresponding computer software. Clean the pipeline with pure acetonitrile solvent, and scan the baseline for UV-vis and near-IR spectra. Use a constant flow pump to circulate the BOP-CuCl_2_ solution through EPR, UV-vis, and near-IR, with a flow rate set to approximately 10 seconds per cycle. Place BOP-CuCl_2_ solution in a low-temperature reactor at 5 °C to cool down. Set software parameters. After the solution cools down, start the three spectrogram tests simultaneously. Start the reaction by adding CHP (0.2 mL) and TTBP solutions at the 2nd and 6th minutes with syringes, respectively. After 100 minutes, the test is completed.

For kinetic studies, we changed the amount of oxidant CHP to investigate the kinetics of the reaction. The other experimental conditions are the same, while the dosage of CHP is 0.1 mL, 0.3 mL, and 0.4 mL, respectively.

*Capture of Cumylperoxyl Radical*:

Bromo-triphenyl-methane capture: Add 0.25 mmol of triphenylmethyl bromide and 0.025 mmol of BOP-CuCl_2_ to a 20 mL sample bottle, dissolve with 2 mL of acetonitrile, and then vacuum and fill them with nitrogen gas. After stirring and cooling to 5 °C, add 50 μL CHP to start the reaction. After about 10 minutes, sample and determine the product using HRMS.

DMPO capture: The experimental steps of the oxidation reaction are the same as the general procedure. Prepare an acetonitrile solution of DMPO with a concentration of 1 mol/L. Take 0.1 mL of reaction solution at different time periods of the reaction, mix it evenly with 0.1 mL DMPO solution, load it into a capillary, and immediately conduct EPR testing.

*In Situ EPR Experiments under Controlled Temperature*:

Prepare an acetonitrile solution of BOP-CuCl_2_ with a concentration of 0.005 mol/L. Add approximately 1cm high BOP-CuCl_2_ solution to a 0.3 mm nuclear magnetic tube, cover it, and cool it to 230 K in the EPR resonant cavity. Test the spectrum. After adding 0.05 mL CHP, immediately cover the lid and conduct tests at 230 K, 250 K, and 270 K to observe whether free radical signals are generated.

*Statistical Analysis*:

The least-squares fittings of EPR spectra were performed in MATLAB using Easyspin 5.1^[1]^ with the garlic function. The kinetic analysis was perfomed using Microsoft Excel.

| **Table S1. Crystal data and structure refinement for BOP-CuCl_2_ complex.** | |
| --- | --- |
|  | |
| Identification code | mo_220511_CuBOP_Cl_0506_0m |
| Empirical formula | C_17_H_23_Cl_2_CuN_3_O_2_ |
| Formula weight | 435.82 |
| Temperature/K | 170.0 |
| Crystal system | orthorhombic |
| Space group | P2_1_2_1_2_1_ |
| a/Å | 11.0266(4) |
| b/Å | 11.1906(4) |
| c/Å | 16.5160(4) |
| α/° | 90 |
| β/° | 90 |
| γ/° | 90 |
| Volume/Å^3^ | 2037.98(11) |
| Z | 4 |
| ρ_calc_g/cm^3^ | 1.420 |
| μ/mm^‑1^ | 1.348 |
| F(000) | 900.0 |
| Crystal size/mm^3^ | 0.49 × 0.41 × 0.4 |
| Radiation | MoKα (λ = 0.71073) |
| 2Θ range for data collection/° | 4.396 to 54.242 |
| Index ranges | -14 ≤ h ≤ 14, -14 ≤ k ≤ 14, -18 ≤ l ≤ 21 |
| Reflections collected | 30929 |
| Independent reflections | 4474 [R_int_ = 0.0261, R_sigma_ = 0.0230] |
| Data/restraints/parameters | 4474/0/230 |
| Goodness-of-fit on F^2^ | 1.073 |
| Final R indexes [I>=2σ (I)] | R_1_ = 0.0172, wR_2_ = 0.0474 |
| Final R indexes [all data] | R_1_ = 0.0178, wR_2_ = 0.0476 |
| Largest diff. peak/hole / e Å^-3^ | 0.23/-0.29 |
| Flack parameter | 0.010(3) |


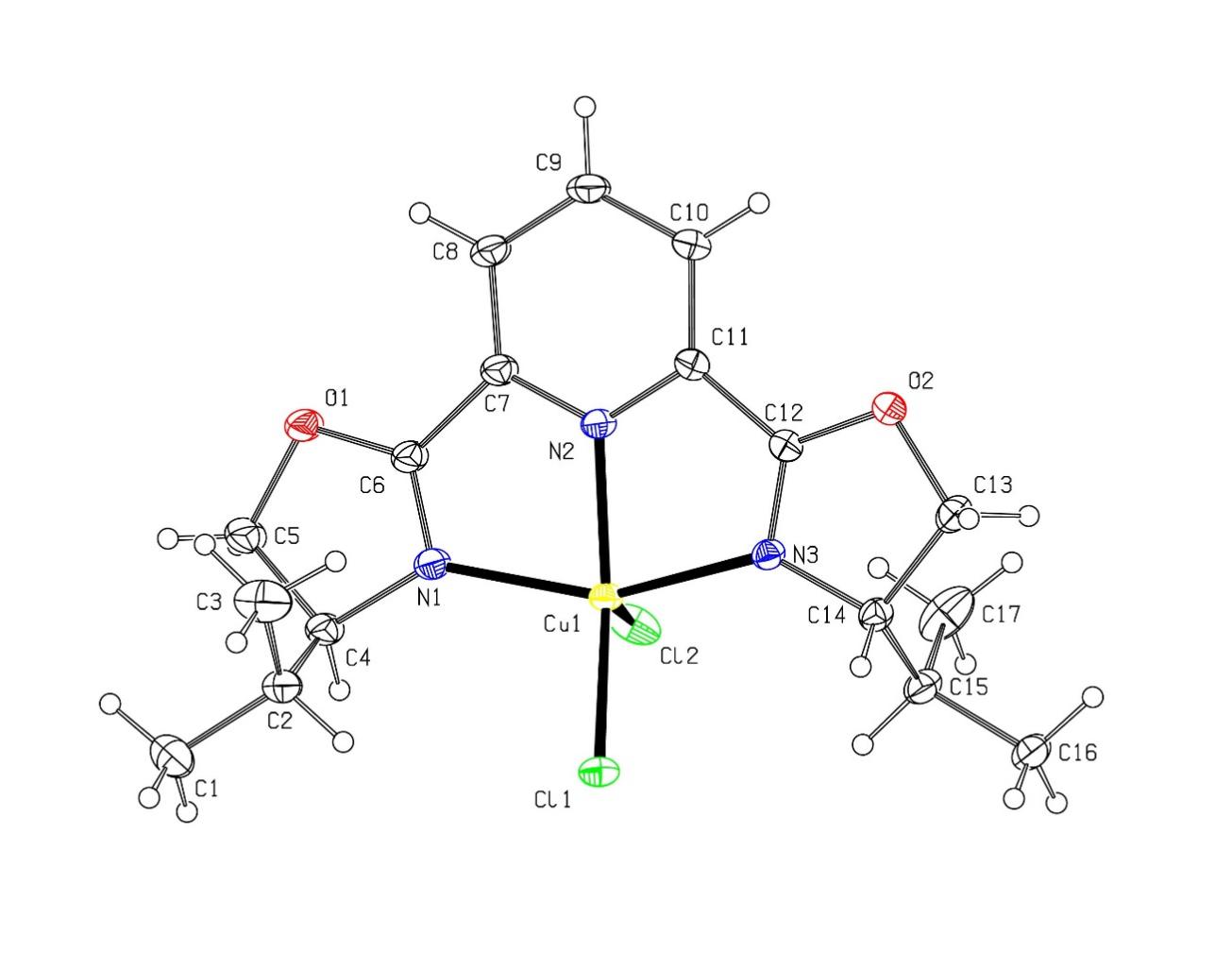


**Figure S3.** ORTEP representation of BOP-CuCl_2_ complex with 30% displacement ellipsoids.

**Figure S4.** EPR spectroscopy (at frequency of 9.8480 GHz). Black: EPR signal of BOP-CuCl_2_; Red: EPR signal after adding TTBP to the solution.

**
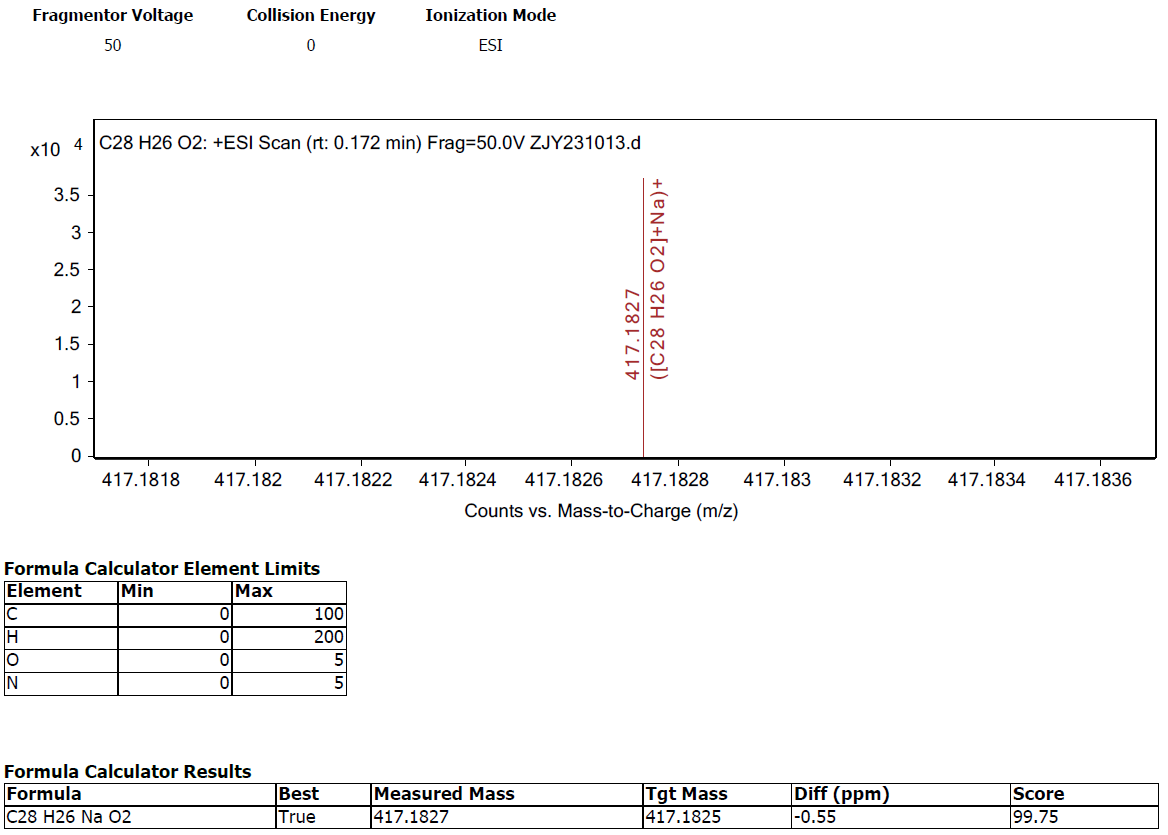
**

**Figure S5.** High-resolution mass spectrometry results for captured cumylperoxyl radical by trityl.


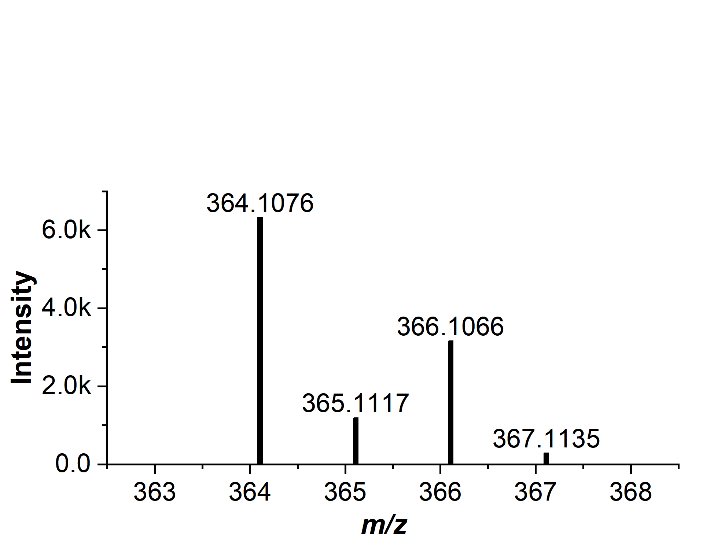


**Figure S6.** High-resolution mass spectrometry results for detecting the CuN_3_^+^ form of LCu(I) in the reaction catalyzed by BOP-CuCl_2_.


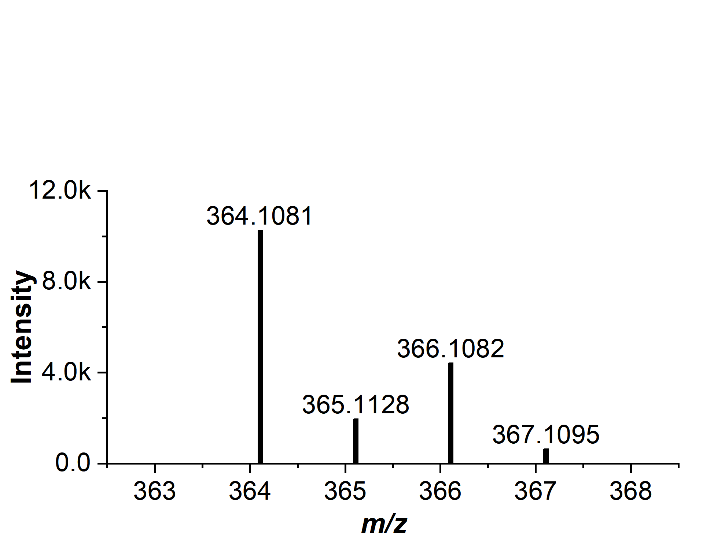


**Figure S7.** High-resolution mass spectrometry results for detecting the CuN_3_^+^ form of LCu(I) after mixing CuCl with BOP ligand.

**
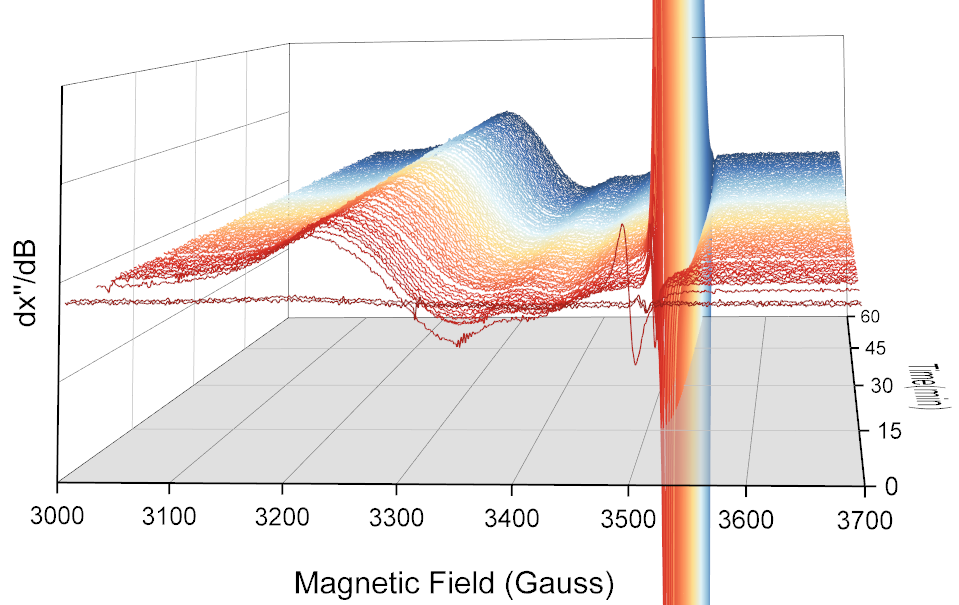
** **
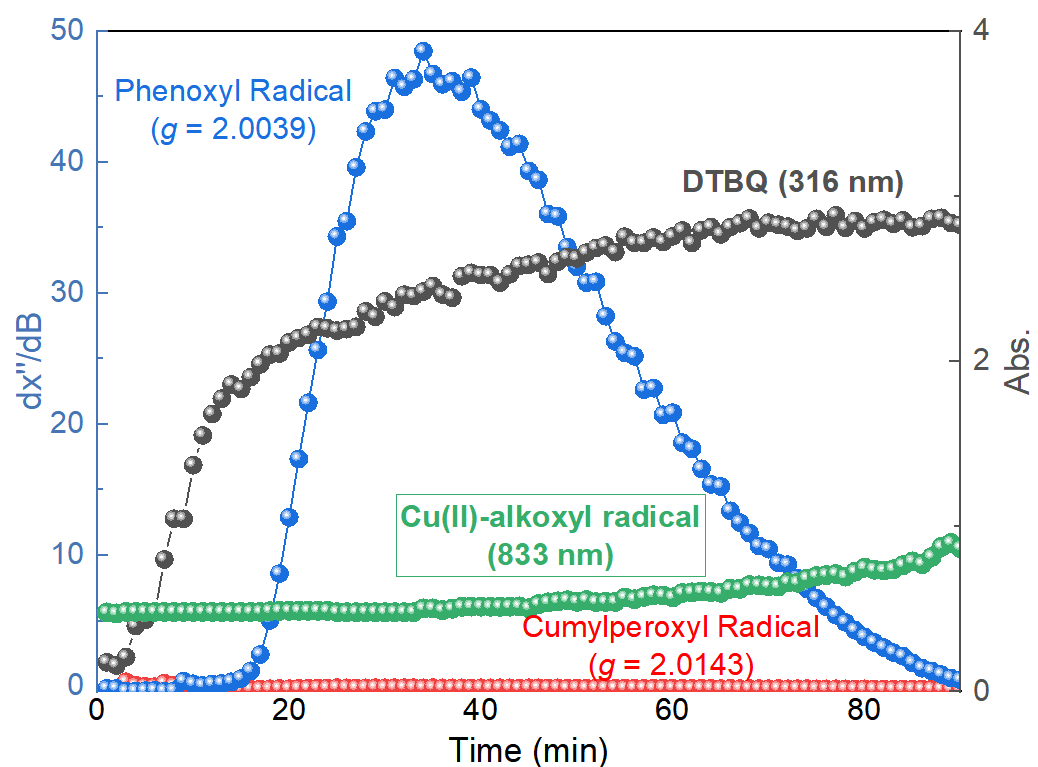
**

**Figure S8.** Left: EPR spectra during the monitoring of Cu(I) initialized reaction. Right: Intensity time course of different observed species for Cu(I) initialized reaction.

**Figure S9.** (a) Scheme of reaction set up. (b) Intensity time course of different observed species for detections with CHP (0.55 mmol, 1.1 equiv) added at 2 min and TTBP (0.5 mmol) at 6 min. Red: Cumylperoxyl Radical; Blue: Phenoxyl Radical; Black: DTBQ; Green: Cu(II)-alkoxyl radical. (c) EPR spectroscopy (at frequency of 9.8488 GHz). (d) near-IR spectroscopy. (e) UV-Vis spectroscopy.

**Figure S10.** (a) Scheme of reaction set up. (b) Intensity time course of different observed species for detections with CHP (1.6 mmol, 3.3 equiv) added at 2 min and TTBP (0.5 mmol) at 6 min. Red: Cumylperoxyl Radical; Blue: Phenoxyl Radical; Black: DTBQ; Green: Cu(II)-alkoxyl radical. (c) EPR spectroscopy (at frequency of 9.8488 GHz). (d) near-IR spectroscopy. (e) UV-Vis spectroscopy.

**
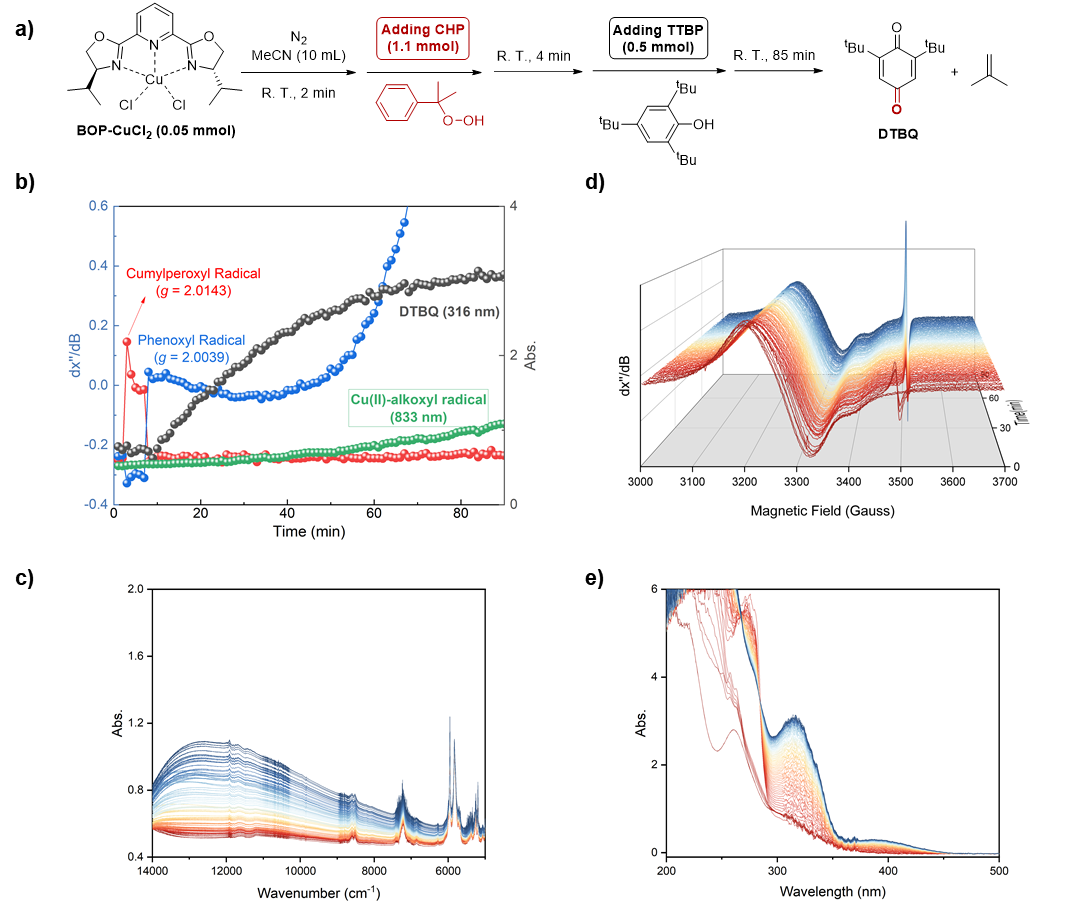
**

**Figure S11.** (a) Scheme of reaction set up at room temperature (23 °C). (b) Intensity time course of different observed species for detections with CHP (1.1 mmol, 2.2 equiv) added at 2 min and TTBP (0.5 mmol) at 6 min. Red: Cumylperoxyl Radical; Blue: Phenoxyl Radical; Black: DTBQ; Green: Cu(II)-alkoxyl radical. (c) EPR spectroscopy (at frequency of 9.8486 GHz). (d) near-IR spectroscopy. (e) UV-Vis spectroscopy.

2. Computational Studies

*Methods*:

DFT calculations were performed in Gaussian 09 D.01.^[2]^ The gas-phase geometries were fully optimized using the BP86-D3 method^[3]^ (with a Becke−Johnson (BJ) damping function^[4]^). The frequency analyses were performed at the same level to confirm that the structure was a minimum, and the computed frequencies were also used to evaluate their zero-point vibrational energy (ZPVE) and thermal corrections at 298 K. Using the gas-phase optimized structures, single-point energies and TD-DFT^[5]^ were obtained at the B3LYP-D3^[3c, 6]^ level with a Becke−Johnson (BJ) damping function,^[4]^ together with the def2-TZVP basis set,^[7]^ including solvation by MeCN under the CPCM model.^[8]^ The g-value calculations for radicals were performed in ORCA 5.0.4 version,^[9]^ at B3LYP-D3^[3c, 6]^ level with def2-TZVP basis set.^[7]^ Computed structures are illustrated using CYLView.^[10]^ The spin density diagrams are illustrated using Chimera.^[11]^

*Cartesian Coordinates and Energies*

G: BP86-D3(BJ)/def2-TZVP Gibbs free energy at 298 K, given in Hartree.

**Cu(II)-(^•^OCumyl)**


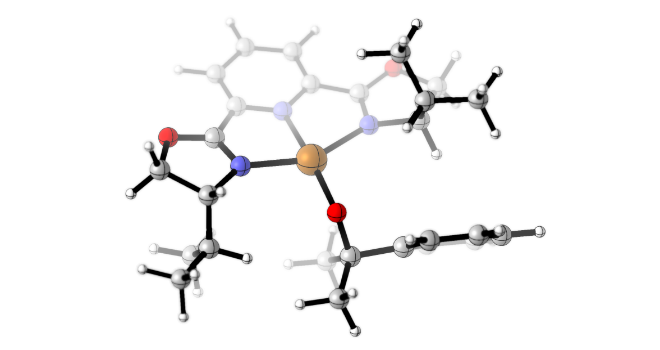


G = -3042.783222

0 imaginary frequencies

----------------------------------------------------------------------

C 2.0721620 2.6621930 -1.0307800

C 1.4294970 1.4337160 -1.1465530

N 0.8379400 0.8714520 -0.0895960

C 0.7957750 1.4710260 1.1041110

C 1.4228280 2.6951850 1.3077490

C 2.0766400 3.2794980 0.2211560

C 1.2028030 0.6039450 -2.3398940

C -0.0591960 0.6945530 2.0137050

O 1.6048260 0.9938400 -3.5391890

C 1.0228440 -0.0190120 -4.4972840

C 0.5008540 -1.1491660 -3.5802540

N 0.5549340 -0.5125470 -2.2310440

N -0.5981080 -0.4097010 1.5988040

C -1.6398560 -0.8159590 2.5853590

C -1.3206430 0.1037100 3.7842090

O -0.3781500 1.1394080 3.2181000

C -3.0502310 -0.6531460 1.9803750

C -3.3565260 0.7790560 1.5317610

C -4.1014190 -1.1732480 2.9690430

C 1.3189020 -2.4532400 -3.6281600

C 1.0810520 -3.1704560 -4.9642820

C 2.8073240 -2.2409540 -3.3419190

Cu -0.0490360 -0.9116990 -0.2951640

H 2.5468140 3.1259430 -1.8932980

H 1.3904890 3.1836400 2.2795660

H 2.5763770 4.2398440 0.3462120

H 0.2417980 0.5118360 -5.0514130

H 1.8443770 -0.3201720 -5.1549170

H -0.5516200 -1.3754910 -3.8008880

H -1.4836610 -1.8701960 2.8464720

H -0.7696420 -0.3820130 4.5963430

H -2.1835700 0.6592320 4.1635620

H -3.0614220 -1.3096910 1.0912140

H -4.3518960 0.8154730 1.0693110

H -2.6300510 1.1408040 0.7869460

H -3.3685580 1.4799200 2.3814380

H -5.0940610 -1.1661730 2.4988690

H -3.8782250 -2.2017730 3.2878460

H -4.1617860 -0.5376560 3.8669420

H 0.8888980 -3.0762680 -2.8258710

H 1.6086130 -4.1343060 -4.9709480

H 0.0109370 -3.3593740 -5.1344170

H 1.4685530 -2.5809240 -5.8095110

H 3.3112220 -3.2130540 -3.2514210

H 2.9748260 -1.6847890 -2.4061120

H 3.3084520 -1.6987400 -4.1588000

O -0.6696360 -2.7000200 -0.4946490

C 0.0695980 -3.8644420 -0.0634790

C -0.1677340 -5.0076670 -1.0528290

H 0.2402790 -5.9373560 -0.6278790

H -1.2355020 -5.1450450 -1.2536640

H 0.3453420 -4.8078850 -2.0030550

C 1.5513810 -3.5424960 0.0924250

H 1.7039280 -2.6828080 0.7664200

H 2.1035340 -4.4025990 0.4984230

H 1.9764040 -3.2965070 -0.8886460

C -0.6570090 -4.0942280 1.2625810

C -0.0022880 -4.0637440 2.5060310

C -2.0523120 -4.3477700 1.2198000

C -0.6958760 -4.3993190 3.6539980

H 1.0619130 -3.8572940 2.5657220

C -2.7470520 -4.6594780 2.3728240

H -2.5657200 -4.3076440 0.2622980

C -2.0702200 -4.6967440 3.5929870

H -0.1727760 -4.4577420 4.6080950

H -3.8121510 -4.8847020 2.3292820

H -2.6046940 -4.9603950 4.5053720

----------------------------------------------------------------------

**Cu(III)-OCumyl**


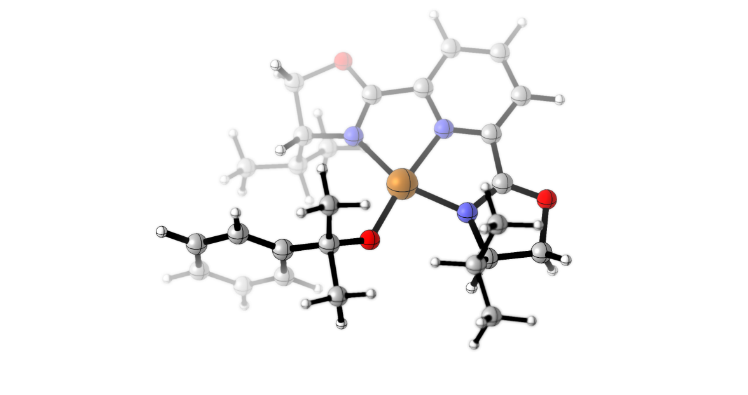


G = -3042.783566

0 imaginary frequencies

----------------------------------------------------------------------

C 1.5517600 2.9644520 -1.6030330

C 1.0722740 1.6597150 -1.5725720

N 0.6872780 1.1097020 -0.4171580

C 0.7522760 1.7584540 0.7475600

C 1.2320400 3.0626640 0.8037860

C 1.6288620 3.6604220 -0.3943510

C 0.8901920 0.6798310 -2.6537280

C 0.2113250 0.8970720 1.8066020

O 1.1809370 0.9380810 -3.9141770

C 0.7717560 -0.3127760 -4.6723050

C 0.5404090 -1.3626950 -3.5621220

N 0.4499750 -0.5032000 -2.3505910

N -0.1998210 -0.3055740 1.5118520

C -0.9704480 -0.8212400 2.6878400

C -0.5346750 0.1538380 3.7986180

O 0.0635780 1.3220910 3.0441050

C -2.4818370 -0.8328890 2.3919490

C -3.0554290 0.5474690 2.0589090

C -3.2213040 -1.4913510 3.5628520

C 1.6661990 -2.4084570 -3.4129370

C 1.6137200 -3.3893390 -4.5925790

C 3.0546750 -1.7881260 -3.2355350

Cu 0.0296860 -0.7606600 -0.4057110

H 1.8535340 3.4251280 -2.5415600

H 1.2825700 3.5988440 1.7493290

H 2.0031680 4.6841950 -0.3865270

H -0.1268150 -0.0349540 -5.2328750

H 1.6089190 -0.5446180 -5.3376030

H -0.4213000 -1.8743530 -3.6966250

H -0.6329020 -1.8429690 2.9047820

H 0.2677690 -0.2201580 4.4434820

H -1.3636610 0.5650400 4.3816930

H -2.5968500 -1.4845700 1.5069080

H -4.1230890 0.4505910 1.8211320

H -2.5635810 1.0020830 1.1839030

H -2.9762870 1.2420330 2.9099640

H -4.2816190 -1.6223820 3.3070260

H -2.7985270 -2.4788520 3.7946880

H -3.1807280 -0.8682750 4.4704420

H 1.4114010 -2.9562870 -2.4905400

H 2.3695830 -4.1755300 -4.4585750

H 0.6262940 -3.8664890 -4.6762910

H 1.8350820 -2.8842380 -5.5454390

H 3.7893610 -2.5817900 -3.0430690

H 3.0903100 -1.0908250 -2.3834090

H 3.3876650 -1.2548200 -4.1396650

O -0.5107620 -2.5219400 -0.6158500

C -0.1364510 -3.6063580 0.1942830

C -0.0560330 -4.8088750 -0.8796020

H 0.1833710 -5.7210890 -0.3222820

H -1.0175120 -4.9108650 -1.3881190

H 0.7355360 -4.5624160 -1.5929380

C 1.2730780 -3.4101490 0.7754760

H 1.2595270 -2.6212540 1.5428510

H 1.6558450 -4.3359150 1.2240550

H 1.9527650 -3.1136380 -0.0344070

C -1.2016920 -4.0359450 1.1647920

C -0.8990040 -4.6305690 2.4000700

C -2.5510450 -3.9627930 0.7551600

C -1.9118490 -5.1330110 3.2019040

H 0.1336860 -4.7159860 2.7329710

C -3.5621770 -4.4583170 1.5582900

H -2.7784620 -3.5248990 -0.2160850

C -3.2450930 -5.0582340 2.7797990

H -1.6709190 -5.6021240 4.1551990

H -4.6001670 -4.4060280 1.2319690

H -4.0348780 -5.4752400 3.4034540

----------------------------------------------------------------------

**Cu(II)(^•^OCumyl)OH**


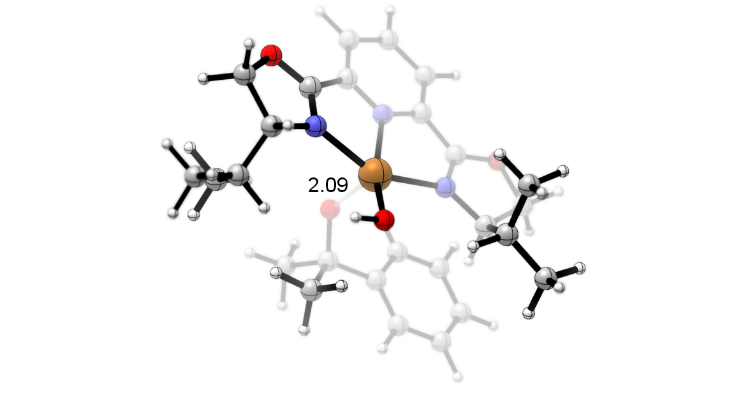


G = -3118.954736

0 imaginary frequencies

----------------------------------------------------------------------

C -0.3457260 3.0673670 -3.2111450

C -0.0698390 1.9074440 -2.4926970

N 0.0272150 1.9393310 -1.1624370

C -0.0981190 3.0710850 -0.4681780

C -0.3685950 4.2754580 -1.1101520

C -0.5050110 4.2548820 -2.4980840

C 0.1771480 0.5449540 -2.9866970

C 0.1481810 2.8195130 0.9596150

O 0.1932610 0.2628320 -4.2938870

C 0.5677820 -1.1963300 -4.3682790

C 0.6255760 -1.6646260 -2.8936600

N 0.4058300 -0.4045640 -2.1505120

N 0.4290270 1.6313360 1.3709560

C 0.6066830 1.6692940 2.8429010

C 0.5316440 3.1844010 3.1511090

O 0.1182340 3.8148270 1.8536000

C -0.4375400 0.7989430 3.5692190

C -1.8742840 1.2367100 3.2757650

C -0.1348850 0.7584300 5.0722050

C 1.9180720 -2.3737800 -2.4550190

C 2.0263850 -3.7263270 -3.1694130

C 3.1697100 -1.5142150 -2.6424280

Cu 0.2702280 0.1885860 -0.1545590

H -0.4316080 3.0388310 -4.2952160

H -0.4651230 5.1978890 -0.5421450

H -0.7217260 5.1794150 -3.0320460

H -0.2208830 -1.6803500 -4.9515210

H 1.5345020 -1.2314770 -4.8832610

H -0.2313990 -2.3177850 -2.6745020

H 1.6112510 1.2798420 3.0670790

H 1.4945640 3.6357960 3.4133070

H -0.2398770 3.4489970 3.8817370

H -0.3010190 -0.2210660 3.1669830

H -2.5750410 0.5635610 3.7905180

H -2.0816960 1.1943510 2.1962750

H -2.0754940 2.2534850 3.6499010

H 0.8914190 0.4118380 5.2683750

H -0.2601840 1.7504270 5.5351110

H -0.8301030 0.0718700 5.5753360

H 1.7886980 -2.5523530 -1.3743710

H 2.8993970 -4.2813850 -2.7986170

H 1.1292450 -4.3397390 -2.9937140

H 2.1520820 -3.5988860 -4.2577450

H 4.0466580 -2.0496870 -2.2533190

H 3.0843940 -0.5635780 -2.0944020

H 3.3680870 -1.2923320 -3.7037760

O 1.0367660 -1.3317960 0.5823710

H 1.1838000 -1.2270640 1.5426210

O -1.8086230 0.0647700 0.0552700

C -2.5086160 -1.1443440 0.0980280

C -2.0121450 -2.0392790 1.2407790

H -2.6631770 -2.9146820 1.3723880

H -0.9783170 -2.3502660 1.0275150

H -2.0284490 -1.4543130 2.1702680

C -3.9866440 -0.6841830 0.4387010

H -4.6165640 -1.5833720 0.4469130

H -3.9888850 -0.2040790 1.4245480

H -4.3407970 0.0129380 -0.3280770

C -2.5378140 -1.8225550 -1.2672000

C -2.7655590 -1.0379950 -2.4030330

C -2.3897290 -3.2004140 -1.4273920

C -2.8412260 -1.6114780 -3.6653770

H -2.8776920 0.0391660 -2.2747750

C -2.4585140 -3.7802960 -2.6938570

H -2.2030710 -3.8354710 -0.5645420

C -2.6892940 -2.9902530 -3.8153500

H -3.0299950 -0.9856710 -4.5377450

H -2.3388860 -4.8579750 -2.7994310

H -2.7626870 -3.4466080 -4.8018310

----------------------------------------------------------------------

***TTBP phenoxyl radical***


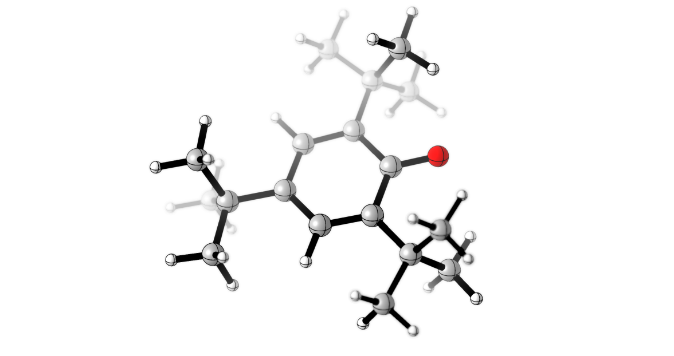


G = -778.720366

0 imaginary frequencies

----------------------------------------------------------------------

C 1.2451730 0.4331720 0.0000540

C 1.1790200 1.8037900 0.0000670

C -1.2395600 1.7886880 -0.0000450

C -1.2744620 0.4096360 -0.0000660

C -0.0088640 -0.3221940 -0.0000230

H 2.1020120 2.3759060 0.0001210

O 0.0012680 -1.5858270 -0.0000420

H -2.1736030 2.3386040 -0.0000790

C -0.0392950 2.5138890 0.0000150

C 2.5803800 -0.3082560 0.0001180

C 2.6847280 -1.1953850 -1.2685510

H 3.6533660 -1.7195470 -1.2606820

H 1.8717200 -1.9286750 -1.2891030

H 2.6353780 -0.5690720 -2.1729090

C 2.6845700 -1.1954500 1.2687570

H 3.6532130 -1.7196030 1.2609870

H 2.6350970 -0.5691840 2.1731410

H 1.8715670 -1.9287490 1.2891680

C 3.7765280 0.6578840 0.0002220

H 3.7814130 1.2975390 -0.8961670

H 3.7812900 1.2974970 0.8966410

H 4.7045950 0.0671520 0.0002720

C -2.5945240 -0.3579440 -0.0001220

C -2.6870540 -1.2464440 -1.2690100

H -1.8660240 -1.9707130 -1.2896070

H -3.6499500 -1.7807730 -1.2608290

H -2.6447010 -0.6193560 -2.1729420

C -2.6870880 -1.2465540 1.2686860

H -3.6499870 -1.7808770 1.2604350

H -1.8660630 -1.9708300 1.2892370

H -2.6447510 -0.6195460 2.1726740

C -3.8083570 0.5862320 -0.0000980

H -3.8261460 1.2251240 0.8963050

H -3.8261170 1.2252040 -0.8964450

H -4.7251070 -0.0220910 -0.0001400

C -0.0055610 4.0406520 0.0000360

C 0.7459000 4.5332880 1.2625490

C 0.7461120 4.5333210 -1.2623390

C -1.4101540 4.6631800 -0.0000750

H 0.2234450 4.2021270 2.1718820

H 1.7747740 4.1495560 1.2935700

H 0.7862610 5.6338580 1.2577310

H 0.2238360 4.2021470 -2.1717690

H 0.7864330 5.6338930 -1.2575050

H 1.7750070 4.1496290 -1.2931760

H -1.3178350 5.7596560 -0.0000530

H -1.9796840 4.3702380 -0.8947200

H -1.9798370 4.3702170 0.8944660

----------------------------------------------------------------------

***Cumylperoxyl radical***


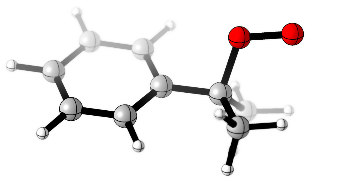


G = -500.100046

0 imaginary frequencies

----------------------------------------------------------------------

O -0.4599440 0.3994230 0.0029430

C -2.3111730 -0.2469660 1.3719460

C -2.0312620 -1.7056690 1.0185180

H -2.5332820 -1.9890030 0.0861300

H -0.9482870 -1.8548850 0.9116750

H -2.4104740 -2.3470960 1.8272240

O -1.7704180 0.6066890 0.2276630

C -1.5609020 0.1774150 2.6255880

H -0.4867060 0.0325350 2.4508840

H -1.7482000 1.2315870 2.8651110

H -1.8646880 -0.4478510 3.4788910

C -3.7877380 0.0901910 1.3546000

C -4.5054260 -0.0408790 0.1613500

C -4.4637790 0.5151420 2.4966450

C -5.8597790 0.2594930 0.1113240

H -3.9889710 -0.3531960 -0.7436590

C -5.8261550 0.7951030 2.4538510

H -3.9305270 0.6259960 3.4369510

C -6.5267770 0.6720210 1.2609030

H -6.3992600 0.1679450 -0.8297620

H -6.3387310 1.1171240 3.3589590

H -7.5902870 0.9002720 1.2245840

----------------------------------------------------------------------

3. References

[1] S. Stoll, A. Schweiger, *J. Magn. Reson.* **2006**, *178*, 42-55.

[2] G. W. T. M. J. Frisch, H. B. Schlegel, G. E. Scuseria, M. A. Robb, J. R. Cheeseman, G. Scalmani, V. Barone, B. Mennucci, G. A. Petersson, H. Nakatsuji, M. Caricato, X. Li, H. P. Hratchian, A. F. Izmaylov, J. Bloino, G. Zheng, J. L. Sonnenberg, M. Hada, M. Ehara, K. Toyota, R. Fukuda, J. Hasegawa, M. Ishida, T. Nakajima, Y. Honda, O. Kitao, H. Nakai, T. Vreven, J. A. Montgomery, Jr., J. E. Peralta, F. Ogliaro, M. Bearpark, J. J. Heyd, E. Brothers, K. N. Kudin, V. N. Staroverov, T. Keith, R. Kobayashi, J. Normand, K. Raghavachari, A. Rendell, J. C. Burant, S. S. Iyengar, J. Tomasi, M. Cossi, N. Rega, J. M. Millam, M. Klene, J. E. Knox, J. B. Cross, V. Bakken, C. Adamo, J. Jaramillo, R. Gomperts, R. E. Stratmann, O. Yazyev, A. J. Austin, R. Cammi, C. Pomelli, J. W. Ochterski, R. L. Martin, K. Morokuma, V. G. Zakrzewski, G. A. Voth, P. Salvador, J. J. Dannenberg, S. Dapprich, A. D. Daniels, O. Farkas, J. B. Foresman, J. V. Ortiz, J. Cioslowski, and D. J. Fox, Revision D.01 ed., Gaussian, Inc., Wallingford, CT, **2013**.

[3] a) A. D. Becke, *Phys. Rev. A* **1988**, *38*, 3098; b) J. P. Perdew, *Phys. Rev. B* **1986**, *33*, 8822; c) S. Grimme, J. Antony, S. Ehrlich, H. Krieg, *J. Chem. Phys.* **2010**, *132*, 154104.

[4] S. Grimme, S. Ehrlich, L. Goerigk, *J. Comput. Chem.* **2011**, *32*, 1456-1465.

[5] a) R. Bauernschmitt, R. Ahlrichs, *Chem. Phys. Lett.* **1996**, *256*, 454-464; b) M. E. Casida, C. Jamorski, K. C. Casida, D. R. Salahub, *J. Chem. Phys.* **1998**, *108*, 4439-4449; c) R. E. Stratmann, G. E. Scuseria, M. J. Frisch, *J. Chem. Phys.* **1998**, *109*, 8218-8224.

[6] a) C. Lee, W. Yang, R. G. Parr, *Phys. Rev. B* **1988**, *37*, 785-789; b) A. D. Becke, *J. Chem. Phys.* **1993**, *98*, 5648-5652.

[7] a) F. Weigend, *Phys. Chem. Chem. Phys.* **2006**, *8*, 1057-1065; b) F. Weigend, R. Ahlrichs, *Phys. Chem. Chem. Phys.* **2005**, *7*, 3297-3305; c) K. Eichkorn, F. Weigend, O. Treutler, R. Ahlrichs, *Theor. Chem. Acc.* **1997**, *97*, 119-124.

[8] a) Y. Takano, K. N. Houk, *J. Chem. Theory Comput.* **2005**, *1*, 70-77; b) M. Cossi, N. Rega, G. Scalmani, V. Barone, *J. Comput. Chem.* **2003**, *24*, 669-681; c) V. Barone, M. Cossi, *J. Phys. Chem. A* **1998**, *102*, 1995-2001.

[9] F. Neese, *Wiley Interdiscip. Rev.: Comput. Mol. Sci.* **2012**, *2*, 73-78.

[10] C. Y. Legault, CYLview v.1.0b ed., Université de Sherbrooke, Canada, **2009**.

[11] E. F. Pettersen, T. D. Goddard, C. C. Huang, G. S. Couch, D. M. Greenblatt, E. C. Meng, T. E. Ferrin, *J. Comput. Chem.* **2004**, *25*, 1605-1612.
